# Relationship between Phobic Anxiety in Work and Leisure Activity Situations, and Optimistic Bias Associated with COVID-19 among South Koreans

**DOI:** 10.3390/ijerph17228436

**Published:** 2020-11-14

**Authors:** Young-Jae Kim, E-Sack Kim

**Affiliations:** Department of Physical Education, Chung-Ang University, Seoul 06974, Korea; yjkim@cau.ac.kr

**Keywords:** COVID-19, South Korean, phobic anxiety, optimistic bias, work, leisure activity

## Abstract

Because of the ongoing COVID-19 pandemic, the public is unable to maintain a proper balance between work and leisure, and an increase in community-based infections is causing severe phobic anxiety. Therefore, the present study investigated the differences in phobic anxiety between work and leisure activities according to optimistic bias among 533 South Korean citizens. Frequency analysis, descriptive statistical analysis, *t*-tests, and a one-way analysis of variance were conducted to examine the data. The results showed that for leisure activities, women showed a higher perception of phobic anxiety. In addition, the group showing high optimistic bias had a higher perception of phobic anxiety in both work and leisure activity situations. Therefore, support measures to lower phobic anxiety among women are needed at the government level, while support and interest from family members are needed at home. Moreover, local governments must ensure active involvement to mitigate phobic anxiety among individuals, and measures are needed to more actively implement infectious disease prevention behaviors.

## 1. Introduction

The current coronavirus disease 2019 (COVID-19) outbreak began in Wuhan, China, in December 2019 [1]. As this virus is very highly contagious, there have been 40,234,290 confirmed cases and 1,121,436 deaths worldwide, and 25,275 confirmed cases and 444 deaths in South Korea, as of 19 October 2020 [2]. The continued increase in confirmed cases has led to the global recognition of the seriousness of the COVID-19 outbreak, which the World Health Organization (WHO) [1] declared a pandemic in March 2020 [3].

In modern society, there is increasing interest in healthy lifestyles, which many people make efforts to achieve [4]. According to Sánchez-Hernández et al. [5], work–life balance (WLB) influences people’s quality of life and health, and quality of life can improve when in harmony with a healthy lifestyle. In other words, high quality of life can reinforce people’s defense mechanisms to cope with stress and provide emotional stability [6]. WLB, which has drawn much research attention in recent years, refers to appropriately balancing social, psychological, and physical aspects between work and leisure in order to reach a satisfactory state [7]. Therefore, participation in various leisure activities that enable people to live a happy and enjoyable life is a very important factor, as it leads not only to psychological stability but also good physical health [8].

With the ongoing spread of COVID-19, South Korea has recently lost approximately 1.55 million jobs, and the number of temporarily laid-off workers has steadily increased to more than 1 million [9]. Moreover, the country has also seen the phenomenon of increased depression prevalence due to social distancing and restrictions on outdoor activities, which represent changes in activities of daily living (ADL) that directly affect individual lives [10]. This change in ordinary life did not happen only in South Korea. The unemployment rate in the United States has exceeded the highest level since the Great Depression owing to the ongoing spread of COVID-19. Thus, anxiety about COVID-19 and depression symptoms are increasing, boosted by financial difficulties and job insecurity [11].

Currently, many people in South Korea are not able to maintain a proper WLB. While the COVID-19 outbreak has led people to spend more time at home with the rise in work from home, cancellations of get-togethers and meetings, and delays in the start of school, they are essentially experiencing forced WLB [12]. This increased time at home has also led to the trend of substituting outdoor leisure activities people previously engaged in with leisure activities that can easily be enjoyed at home [13]. However, people are still enjoying leisure activities, despite various codes of conduct established to prevent the spread of a highly infectious disease. Many people continue to use crowded places such as computer rooms, cafes, and restaurants, leading to increased fear of infection, and phobic anxiety is being recognized [14]. Anxiety and fear related to the spread of COVID-19 have led to changes in personal lifestyles or ADLs, and people are facing new situations and issues on which they would not normally focus, such as public health and infectious diseases, as these have emerged as issues that restrict their ADLs [15]. From this point of view, phobic anxiety is characterized by excessive and irrational fear of specific targets or situations [16], leading people to experience a state of panic when confronted with fear-evoking stimuli [17].

Thus, the COVID-19 outbreak has created great phobic anxiety about infection, in the aftermath of infectious diseases like the Spanish flu, severe acute respiratory syndrome (SARS), and Ebola [18]. People may feel even greater phobic anxiety owing to more confirmed cases, deaths, and continued outbreaks than what they experienced prior to COVID-19 [19]. In particular, in Daegu, South Korea, there have been 7155 confirmed cases and 196 deaths; as of 29 October 2020 [20], Daegu is the city with the second-highest number of confirmed cases, following Wuhan, China, where COVID-19 originated [21]. Recently, people in South Korea have experienced greater fear of infection associated with COVID-19 owing to outbreaks in areas other than Daegu, such as cluster infections that occurred in Itaewon and at the distribution centers in the Seoul and Gyeonggi regions [22]. Therefore, investigating current phobic anxiety associated with COVID-19 among South Koreans owing to its continued spread could enable the establishment of measures aiming to reduce incurred emotional trauma and consequently help people regain a healthy lifestyle.

De Zwart et al. [23] reported that, during an infectious disease outbreak such as SARS, people with high levels of optimistic bias tend to be less likely to perceive themselves as vulnerable to infectious diseases, compared to people with lower levels of such bias. In this context, optimistic bias can be defined as an incorrect belief that one is less likely to experience a negative event than others, when evaluating the risk of that negative event occurring [24]. Furthermore, optimistic bias applies to people who think their risk level may be lower than that of other people, based on judgments they or another person have made [25,26]. Moreover, Park et al. [27] also reported that people with high optimistic bias perceived themselves as less likely to be infected than others. According to Lee [28], South Koreans aged 20–39 years tend to believe that COVID-19 is “a disease that you can get if you are unlucky” and show high optimistic bias, believing that “the infection itself cannot be prevented no matter how careful you are.” Additionally, Madani et al. [29] reported that men have greater optimistic tendencies than women in relation to being infected by COVID-19, whereas women show higher phobic anxiety associated with infection and practice preventive hygiene measures more thoroughly than men.

Accordingly, the present study aimed to identify the differences in the level of phobic anxiety perceived at work (or school) and during leisure activities, according to the level of optimistic bias South Koreans perceive during the COVID-19 outbreak. The present study will provide data which should be considered when designing interventions aimed at overcoming emotional difficulties by raising awareness of infectious diseases and recommending infection prevention behaviors in the event of future viral outbreaks.

## 2. Materials and Methods

### 2.1. Study Design

The present study used a cross-sectional design employing self-report questionnaires to investigate levels of phobic anxiety and optimistic bias associated with COVID-19 experienced among South Koreans during work and leisure activities. Data for the study were collected over a 5-day period (Seoul, South Korea 31 July–4 August 2020).

### 2.2. Participants

This study targeted general office workers in South Korea, aged 19–65 years. Participants were selected using convenience sampling, a non-probability sampling method. Embrain, a professional research company, distributed 533 questionnaires to the participants through email and on the company’s website. Prior to answering the questionnaire, all participants provided informed consent by answering a question at the beginning of the survey asking if they consented to participate in the study. The size of the sample necessary for this study was based on the standards defined by Comrey [30] (i.e., 100 = bad, 200 = normal, 300 = good, 500 = very good, 1000 = excellent). Accordingly, 533 samples were used for this study. Since the survey contained questions regarding COVID-19 and phobic anxiety, the study was conducted after receiving approval from the Institutional Review Board of Chung-Ang University (1041078-202007-HRSB-171-01).

### 2.3. Measurements

In the present study, a questionnaire was used to examine phobic anxiety among South Korean people during the COVID-19 outbreak (Seoul, South Korea 31 July–4 August 2020) and analyze differences between phobic anxiety in work and leisure activities, according to level of optimistic bias. This questionnaire comprised items on the following demographic characteristics: gender (male, female), age (20–29, 30–39, 40–49, 50–59, 60–65), marital status (single, married), occupation (managers; professionals and related; service workers; skilled agricultural, forestry, and fishery workers; craft and related trade workers; equipment, machine operating and assembly workers; unskilled laborers), and average monthly income (<₩1,000,000, ₩1,010,000–2,000,000, ₩2,010,000–4,000,000, ₩4,010,000–6,000,000, ≥₩6,010,000) based on the 2019 National Leisure Activities Survey [31] by the Ministry of Culture, Sports and Tourism in South Korea. In addition, there were questionnaire items on phobic anxiety (7 work questions and 7 leisure activity questions), optimistic bias (2 questions), and types of leisure activities (8 questions).

#### 2.3.1. Phobic Anxiety

To measure phobic anxiety among people in South Korea during the COVID-19 pandemic, this study applied the Korean version of the Symptom Checklist-90-Revision (SCL-90-R) that was originally developed by Derogatis [32], translated and revised by Kim et al. [33], to help Korean people understand it easily, and has validity and reliability re-verified by Korean people. We also re-verified the reliability of the Korean-version SCL-90-R [31] within our present sample. The SCL-90-R is a self-report measure comprising 90 common psychopathology items and has been recognized for its ability to differentiate among patients with different types of psychological disorders, including depression and psychological anxiety [34]. It includes 9 sub-symptom domains: somatization, obsessive-compulsive behavior, interpersonal sensitivity, depression, anxiety, hostility, phobic anxiety, paranoid ideation, and psychoticism. Each item represents one psychological symptom of each disorder. Responses are rated on a 5-point Likert-type scale, ranging from 1 (never) to 5 (always). In terms of scoring phobic anxiety, which is a subfactor of SCL-90-R, a total of 17 points or over in all the questions indicated psychological abnormality.

Although the SCL-90-R has 9 subfactors, only the “phobic anxiety” subfactor was used in the present study. This subfactor consists of 7 questions in total; however, for our study, we revised and adapted the measure to be applied twice, once for phobic anxiety in work and once for phobic anxiety in leisure activities. Thus, our questionnaire included 14 items measuring phobic anxiety, with 7 items each for both situations examined in the present study. Cronbach’s alpha for phobic anxiety was 0.79 in the study by Kim et al. [33]. Prior to the reliability testing, the confidence level for verifying the optimum number of reliable samples was set at 95%. In relation to the population size, this study targeted 533 participants, and the margin of error was 5%. Finally, 224 samples were extracted from this setting. In the present sample, the Cronbach’s alpha values were 0.931 and 0.930 for work and leisure activities, respectively.

#### 2.3.2. Optimistic Bias

To measure optimistic bias, the present study used the standard measurement tool developed by Weinstein [25]. This scale was created after consultation with two experts, a professor and a doctorate holder, both of whom specialized in the sociology of sports. Construct validity was confirmed, and the scale was revised to be used in the context of the COVID-19 outbreak. This scale included the two following questions: “What do you believe is the likelihood of someone other than yourself getting COVID-19?” and “What do you believe is the likelihood of you getting COVID-19?” Each question was rated on a 7-point Likert scale (1: it is absolutely unlikely to occur; 2: it is highly unlikely to occur; 3: it is a little unlikely to occur; 4: it is somewhat likely to occur; 5: it is a little likely to occur; 6: it is highly likely to occur; 7: it is very highly likely to occur.) Scores for each question range from 1 to 7 points, and a mean score of the two questions of 4 (median) or below indicates no optimistic bias, while mean scores higher than 4 indicate high levels of optimistic bias. Cronbach’s alpha for optimistic bias was 0.856 in the present sample.

#### 2.3.3. Types of Leisure Activities

The types of leisure activities measured in the present study were based on the classification of leisure activity types used in the 2019 National Leisure Activities Survey [31]. To determine which leisure activities people were participating in during the COVID-19 outbreak, the respondents could make multiple selections. Leisure activities were divided into eight types: (1) viewing cultural and artistic events (*n* = 135, 25.3%; attending exhibitions, music concerts, operas, plays/ballet, musicals, and movies); (2) participating in cultural and artistic events (n = 38, 7.1%; participating in literary events, writing, reading discussions, art activities, playing instruments/singing class, photography, dance); (3) viewing sports (n = 98, 18.4%; in-person viewing: visiting stadiums to view soccer, baseball, volleyball, and other games); other viewing: Television (TV) and Digital multimedia broadcasting (DMB); viewing online games); (4) participating in sports (n = 125, 23.5%; badminton, golf, swimming, aerobics, jogging, bowling, table tennis); (5) travel (n = 159, 29.8%; visiting cultural sites, camping, overseas travel, regional festivals, amusement parks); (6) hobbies (n = 248, 46.5%; collection activities, cooking, hiking, fishing, reading); 7) rest (n = 465, 87.2%; strolling, walking, bathing, sauna, napping, TV watching, listening to the radio, listening to music, subscription to newspapers/magazines, doing nothing); and (8) social activities (n = 465, 87.2%; social volunteer work, religious activities, clubs and nightclubs, visiting family, telephone conversations, meeting friends).

### 2.4. Data Analysis

To achieve the objectives of the present study, SPSS version 25.0 was used to perform all analyses after coding and data cleaning. First, Cronbach’s ɑ levels were tested to ensure the reliability of the measurement tools. Frequency analysis, descriptive statistical analysis, *t*-tests, and one-way ANOVAs were performed to compare the differences in phobic anxiety according to demographic factors. Skewness and kurtosis values were confirmed based on West et al. [35]. Skewness ranged from 0.30 to 0.71, and kurtosis ranged from 0.54 to −1.49, indicating normal distribution. We verified effect sizes according to the values outlined by Cohen [36] (d = 0.2, 0.5, and 0.8 represent small, medium, and large effect sizes, respectively). Lastly, a *t*-test was performed to identify the differences in phobic anxiety according to optimistic bias in work and leisure activities.

## 3. Results

Table 1 shows the demographic characteristics of the study participants. There were a similar number of female (*n* = 269, 50.5%) and male (*n* = 264, 49.5%) participants. With respect to age groups, the highest number of participants were aged between 40 and 49 years (*n* = 134, 25.1%), followed by 20–29 years (*n* = 132, 24.8%), 30–39 years (*n* = 130, 24.4%), 50–59 years (*n* = 125, 23.5%), and 60–65 years (*n* = 12, 2.3%). Among the demographic characteristics, a significant gender difference was found for phobic anxiety in leisure activities.

Table 2 shows the results of the independent sample *t*-test performed to analyze the differences in phobic anxiety in work and leisure activity situations according to optimistic bias among South Koreans during the COVID-19 outbreak. The results showed significant differences for both work and leisure activity situations. In particular, the group with high optimistic bias showed high phobic anxiety in work (M = 3.28, SD = 1.12) and leisure activity (M = 3.44, SD = 1.05) situations on items, related to the place those activities were performed. Phobic anxiety in work (M = 2.25, SD = 1.12) and leisure activity (M = 2.47, SD = 1.18) situations were lowest when participants were alone at a specific place.

Table 3 shows the results of independent sample *t*-tests performed to determine whether there were differences in optimistic bias perceived by South Koreans during the COVID-19 outbreak. The results indicated that the group with a high optimistic bias showed differences in phobic anxiety in work (M = 2.85, SD = 0.90) and leisure activity (M = 3.12, SD = 0.87) situations.

## 4. Discussion

The present study aimed to investigate levels of phobic anxiety associated with COVID-19 perceived by South Koreans, and identify the differences in the level of phobic anxiety in work and leisure activity situations, according to the level of optimistic bias.

First, phobic anxiety associated with COVID-19 in leisure activities among South Koreans was higher in female than in male participants. According to Chol [37], women often feel more burdened when at home, owing to responsibilities related to housework and childcare. As schools and kindergartens have been shut down since the start of the COVID-19 outbreak, many women have had fewer opportunities to participate in leisure activities, thus leading them to feel more depressed, anxious, and afraid, compared with men [38,39]. Furthermore, findings by Reisch et al. [40] implied that women avoid visiting recreational locations, because they worry about being infected with COVID-19, while men visit these places more than women. Accordingly, it can be considered that women have higher levels of fear related to COVID-19 than men.

Furthermore, according to Henriques [41], women are more sensitive to COVID-19 and have higher phobic anxiety associated with COVID-19 than men. This is consistent with the findings of the present study in that women could be considered to view and accept COVID-19 more sensitively than men. Moreover, Duarte [42] suggested that, compared with men, women care more about preventing the spread of COVID-19 through infection prevention behaviors, such as mask-wearing and hand-washing, as was also the case during the 2002 SARS outbreak. In line with the findings of the present study, Capraro and Barcelo [43] reported that men described mask-wearing as “embarrassing, not cool, and symboliz[ing] weakness,” and believed that they were less likely to become infected with COVID-19 than women. Taken together, these findings indicate that men have lower phobic anxiety associated with COVID-19 than women. Therefore, it is believed that national-level programs to improve infection prevention behavior awareness are needed to further raise the awareness of infectious disease risk among men. Moreover, other measures to decrease the level of phobic anxiety associated with COVID-19 through continued interest and improvement from the government and society are needed to reduce the trauma associated with COVID-19 and promote mental health among women.

Second, the present results on optimistic bias showed that the group with high optimistic bias had higher levels of phobic anxiety associated with COVID-19 in both work and leisure activity situations. Zhong et al. [44] reported that Chinese participants exhibited an optimistic attitude toward COVID-19 and were confident that they could win the war against the virus. However, as the news and other forms of media instilled fear of COVID-19, they became more cautious of crowded places and wore a mask when leaving the house. This could be viewed as having a low perception of optimistic bias at a personal level, but strictly complying with infection prevention behavior due to continuously rising awareness of the dangers at the national level. In other words, wearing a mask in one’s daily life or avoiding crowded places could be viewed as strictly complying with infection prevention behaviors. Even in South Korea, wearing a mask or avoiding crowded places represents practicing prevention behaviors for protecting oneself. Accordingly, it is determined that people who comply with such prevention behaviors would have higher phobic anxiety. Kim [45] found that, owing to the fear of COVID-19 infection, people have changed their habits regarding visiting cultural facilities or nearby travel destinations to enjoy leisure activities. Further, compared to before the COVID-19 outbreak, visits to places where crowds of people are likely, such as science museums, theaters, and art galleries, have decreased by 60%, whereas visits to drive-in theaters have increased by 112%. Thus, based on these previous findings as well as those of the present study, it appears that people still enjoy leisure activities; however, they prefer to engage in such activities while refraining from contact with others as much as possible, because they feel anxious about being infected with COVID-19 when visiting crowded places.

In Daegu, South Korea, people began to experience anxiety associated with COVID-19 infection owing to an exponential increase in confirmed cases starting with the Shinchunji Church cluster [46]. Such a cluster outbreak increases people’s fear of gathering in crowded places, resulting in an increased number of people who avoid meeting others for personal reasons [47]. However, despite such prevention behaviors, several confirmed COVID-19 cases were reported to have originated at the Itaewon club in Seoul [48], and a cluster outbreak of COVID-19 also occurred at a delivery distribution center in Bucheon Coupang [49]. This resulted in people experiencing phobic anxiety associated with contracting COVID-19 through contact with others they do not know, even if they strictly comply with prevention behaviors.

Nyenhuis et al. [50] reported that individuals in England, Germany, and the United States have a low optimistic bias associated with the possibility of becoming infected with COVID-19. It is believed that optimistic bias was lowered by reducing social interactions and strictly complying with personal hygiene rules. Moreover, Plomecka et al. [51] suggested that continued delivery of a variety of information about COVID-19 through social networking services, mass media, and communication with nearby acquaintances could lower optimistic bias.

Furthermore, the subway in Seoul is very congested during commute hours, to the point of being referred to as “hell subway,” and people get on and off the trains in very crowded environments. Accordingly, the South Korean government established a measure to prevent people from boarding subway trains without wearing masks [52]. To avoid subway overcrowding, companies have implemented shift work and selective work systems. However, people still fear the subway, because congestion remains in 14 subway stations in the Yeouido, Jong-ro, and Gangnam districts of Seoul, where large corporations are concentrated [53]. Therefore, to successfully handle various psychosocial problems, the government, medical personnel, and other stakeholders must focus on prevention behaviors and intervention programs. Moreover, with respect to raising awareness of infectious diseases and increasing prevention behaviors, active government interventions should focus on reaching people through the Internet, mobile phones, and social media.

This study had the following limitations. First, the study was conducted online through a professional research company, because it was impossible to distribute questionnaires in person during the consistent spread of COVID-19 and social distancing. Therefore, respondents’ ways and skills of using computers might have influenced their ways of responding. Second, this was a cross-sectional study, which means that respondents’ phobic anxiety might have been affected by social circumstances when the study was conducted and individual psychological factors. As the COVID-19 pandemic continues, a longitudinal study should be conducted to investigate phobic anxiety caused by COVID-19 in more detail. Third, the study results cannot be generalized and applied to other countries and groups outside of South Korea. Fourth, this study did not examine the level of phobic anxiety recognized by the participants before taking part in this study. The participants’ pre-existing thoughts, situations, and conditions might have been different. Therefore, further research will need to control for varied situations and conditions of participants to obtain more meaningful results. Fifth, in this study, further clinical assessment was not conducted for participants with high scores of phobic anxiety about COVID-19, as we used convenience sampling and non-probability sampling. Further research including clinical assessment when the SCL-90-R is used would yield more meaningful inquiry results.

## 5. Conclusions

The present study investigated phobic anxiety in work and leisure activities among South Koreans during the COVID-19 outbreak and identified differences in phobic anxiety according to individuals’ optimistic bias. Based on the findings, the following conclusions were derived.

Women showed higher phobic anxiety in leisure activities than men. With respect to phobic anxiety according to optimistic bias, the group with high optimistic bias showed higher levels of phobic anxiety in both work and leisure activity situations. This can be viewed as men having less fear of COVID-19 than women, and women demonstrating higher phobic anxiety in leisure activities owing to less time spent on leisure activities as a result of the increased burden of housework and childcare, along with employment anxiety experienced during the COVID-19 outbreak. Therefore, a national-level program should be developed and disseminated to raise awareness of COVID-19 among men. Additionally, the country should develop programs for women in need so they can get over the COVID-19 situation, and maintaining jobs is needed for women. Moreover, men and women should share housework and childcare responsibilities at home, thus allowing women to find some joy during the current COVID-19 pandemic. Moreover, men need to develop plans to help women who are having a difficult time owing to COVID-19, either at home or at work.

Moreover, the findings showed that the group with high optimistic bias also had higher levels of phobic anxiety. Therefore, local governments should take measures to reduce phobic anxiety in individuals when national disasters, such as COVID-19, occur and find methods to encourage more active participation in infectious disease prevention behaviors.

## Figures and Tables

**Table 1:** Demographic characteristics (*n* = 533).

Variable	*n*	%	Work (Phobic Anxiety)			Leisure Activities (Phobic Anxiety)		
			M	SD	*p*-Value	M	SD	*p*-Value
Gender								
Male	264	49.5	2.34	0.89	0.246	2.49	0.90	0.006
Female	269	50.5	2.43	0.90		2.71	0.90	
Age, Mean (SD)	40.09 (10.79)							
20–29	132	24.8	2.29	1.00	0.220	2.50	0.99	0.228
30–39	130	24.4	2.34	0.78		2.54	0.76	
40–49	134	25.1	2.51	0.86		2.72	0.90	
50–59	125	23.5	2.42	0.94		2.62	0.96	
60–65 years	12	2.3	2.21	0.79		2.70	1.02	
Marital Status								
Single	245	46.0	2.33	0.92	0.203	2.52	0.91	0.069
Married	288	54.0	2.43	0.87		2.67	0.91	
Job								
Managers	144	27.0	2.36	0.84	0.198	2.62	0.84	0.187
Professionals and related	177	33.2	2.45	0.89		2.68	0.93	
Service workers	125	23.5	2.38	0.91		2.57	0.91	
Skilled agricultural, forestry, and fishery workers	4	0.8	1.25	0.24		1.60	0.66	
Craft and related trades workers	24	4.5	2.46	0.91		2.68	0.90	
Equipment, machine operating, and assembly workers	17	3.2	2.19	1.11		2.30	1.05	
Unskilled laborers	42	7.9	2.45	0.98		2.51	0.93	
Average monthly income								
Under ₩1,000,000	10	1.9	2.70	0.85	0.236	2.77	0.79	0.225
₩1,010,000–2,000,000	83	15.6	2.22	0.92		2.39	0.88	
₩2,010,000–4,000,000	286	53.7	2.34	0.91		2.62	0.92	
₩4,010,000–6,000,000	103	19.3	2.43	0.85		2.64	0.89	
₩≥ 6,010,000	51	9.6	2.52	0.88		2.70	0.95	

Notes: Mean and standard deviation values were derived by performing a *t*-test and a one-way ANOVA (*p* < 0.05).

**Table 2:** Analysis of differences in phobic anxiety according to optimistic bias (*n* = 533).

Item	Mean (SD)		Effect Size	*t-*Value	*p*-Value
	Low Optimistic Bias (*n* = 356)	High Optimistic Bias (*n* = 177)			
Work (phobic anxiety)					
Q1. Since COVID-19, I am afraid whenever I go to my place of work (school).	2.06 (0.85)	2.81 (1.05)	−0.785	−8.842	0.000
Q2. Since COVID-19, I am afraid to leave the house alone to go to work (school).	1.74 (0.88)	2.35 (1.11)	−0.609	−6.900	0.000
Q3. Since COVID-19, I am afraid to use public transportation to go to work (school).	2.49 (1.05)	3.24 (1.12)	−0.688	−7.522	0.000
Q4. Since COVID-19, I avoid work (school)-related objects, places, or actions because of fear.	2.16 (0.99)	2.85 (1.19)	−0.627	−7.047	0.000
Q5. Since COVID-19, I feel uncomfortable when many people gather at my place of work (school).	2.56 (0.99)	3.28 (1.11)	−0.681	−7.582	0.000
Q6. Since COVID-19, I feel unsettled or scared when left alone at my place of work (school).	1.74 (0.94)	2.25 (1.12)	−0.492	−5.588	0.000
Q7. Since COVID-19, I worry about becoming infected at my place of work (school).	2.36 (0.96)	3.23 (1.15)	−0.819	−9.189	0.000
Leisure activity (phobic anxiety)					
Q1. Since COVID-19, I am afraid to go to places for leisure activities.	2.50 (0.89)	3.24 (1.07)	−0.751	−8.515	0.000
Q2. Since COVID-19, I am afraid to leave the house alone to go participate in leisure activities.	1.87 (0.91)	2.60 (1.21)	−0.683	−7.850	0.000
Q3. Since COVID-19, I am afraid to use public transportation to go participate in leisure activities.	2.57 (1.05)	3.31 (1.09)	−0.692	−7.479	0.000
Q4. Since COVID-19, I have avoided leisure activity-related objects, places, or actions because of fear.	2.44 (1.03)	3.28 (1.07)	−0.796	−8.702	0.000
Q5. Since COVID-19, I feel uncomfortable when I go to a place that has many people for leisure activities.	2.66 (0.95)	3.44 (1.05)	−0.775	−8.552	0.000
Q6. Since COVID-19, I feel unsettled or scared when left alone at places for leisure activities.	1.81 (0.99)	2.47 (1.18)	−0.605	−6.727	0.000
Q7. Since COVID-19, I worry about becoming infected in places where I participate in leisure activities.	2.57 (0.97)	3.50 (1.08)	−0.903	−9.952	0.000

Notes: *p*-values were derived using an independent samples *t*-test; *p* < 0.000 was found in all questions.

**Table 3:** Analysis of differences according to optimistic bias (*n* = 533).

Items	Mean (SD)		Effect Size	*t-*Value	*p*-Value
	Low Optimistic Bias (*n* = 356)	High Optimistic Bias (*n* = 177)			
Work (Phobic Anxiety)	2.16 (0.80)	2.85 (0.90)	−0.822	−9.117	0.000
Leisure Activities (Phobic Anxiety)	2.35 (0.82)	3.12 (0.87)	−0.917	−10.069	0.000

Notes. *p*-values were derived using an independent samples *t*-test.
